# Assessing metabolic flexibility in adults under physiological conditions: Effects of dietary fat and exercise in whole‐room calorimetry

**DOI:** 10.14814/phy2.70979

**Published:** 2026-06-19

**Authors:** Carmen P. Ortega‐Santos, Edward L. Melanson, Daniel H. Bessesen, Pan Zhaoxing, Corey A. Rynders, Audrey Bergouignan

**Affiliations:** ^1^ Exercise and Nutrition Sciences, Milken Institute School of Public Health The George Washington University Washington DC USA; ^2^ Division of Endocrinology, Metabolism, and Diabetes, Department of Medicine, Anschutz Health and Wellness Center University of Colorado‐Anschutz Medical Campus Aurora Colorado USA; ^3^ Division of Geriatric Medicine, Department of Medicine University of Colorado Anschutz Medical Campus Aurora Colorado USA; ^4^ Denver Health Medical Center Denver Colorado USA; ^5^ Department of Biostatistics University of Colorado School of Medicine Aurora Colorado USA; ^6^ Institut Pluridisciplinaire Hubert Curien Université de Strasbourg, CNRS Strasbourg Strasbourg France; ^7^ UMR 7178 Centre National de la Recherche Scientifique (CNRS) Strasbourg France

**Keywords:** exercise, high‐fat diet, insulin, metabolic flexibility, obesity, respiratory quotient, substrate oxidation, weight reduction, whole room calorimeter

## Abstract

Metabolic flexibility (MetaFlex) reflects the ability to adjust substrate oxidation to changes in fuel availability and energy demand. How MetaFlex should be assessed under physiologically relevant metabolic challenges remains unclear. Data from two studies were analyzed in adults with contrasting adiposity and training status, known to influence MetaFlex: healthy lean inactive or trained adults (Ln‐inactive/Ln‐trained, BMI: 19–25 kg/m^2^), inactive adults with obesity (Ob‐Inactive, BMI: 30–40 kg/m^2^), and weight‐reduced inactive or trained (RO‐inactive/RO‐trained, BMI: 25‐40 kg/m^2^, >10% weight loss). Participants completed 24‐h stays in a whole‐room calorimeter under a low‐fat (20% fat, LF‐EB), high‐fat (50% fat, HF‐EB) diet, or acute moderate‐intensity exercise (EX‐EB) in energy balance, or resting in energy balance (REST‐EB), or acute exercise inducing 15% energy deficit (EX‐ED). MetaFlex was assessed using day‐night respiratory quotient (ΔRQ_day‐night_), nighttime RQ (RQ_sleep_) and 24‐h variances of RQ and insulin. Linear mixed models were used. Compared with LF‐EB, HF‐EB increased ΔRQ (Difference LSMeans ± SE, 0.040 ± 0.016, *p* = 0.02) and 24‐h RQ variance (0.895 ± 0.345, *p* = 0.02) and reduced 24‐h insulin variance (0.65 ± 0.13, *p* < 0.001) across groups. EX‐EB did not modify any MetaFlex index. During EX‐ED, 24‐h insulin variance differed by group, with higher values in Ob‐Inactive compared with Ln‐Inactive, RO‐Trained, and RO‐Inactive (*p* < 0.022 for all). MetaFlex is challenge‐dependent. When EB is maintained, short‐term high‐fat feeding is more effective than acute exercise for probing MetaFlex. Exercise combined with an energy deficit reveals between‐group differences driven primarily by insulin dynamics. Individuals with obesity display reduced MetaFlex, whereas weight loss—especially when combined with training—partially restores MetaFlex.

## 1 INTRODUCTION

Metabolic inflexibility, or impaired metabolic flexibility (MetaFlex), refers to the reduced ability of the body to appropriately adjust fuel use in response to changes in substrate availability and energy demand. Originally defined by Kelley and Mandarino in the early 1990s [1], MetaFlex was operationalized as the capacity to switch from predominant lipid oxidation during fasting to glucose oxidation under insulin‐stimulated conditions, typically measured by postprandial changes in respiratory quotient (ΔRQ). Impaired MetaFlex has since been associated with obesity, insulin resistance, and type 2 diabetes, whereas greater flexibility is considered a hallmark of metabolic health (Kelley & Mandarino, 2000; Pannacciulli et al., 2007).

Despite its physiological relevance, how MetaFlex should be defined and measured remains debated. Metabolic inflexibility is often used as a descriptive, but not formal, threshold defined in the literature, which contributes further to the introduction of MetaFlex to clinical practice. As highlighted by Galgani and Fernández‐Verdejo (2021), improvements in MetaFlex after weight loss through lifestyle interventions, such as exercise training (Dubé et al., 2012; Goodpaster et al., 2003) or dietary restrictions (Kelley et al., 1999), often disappear after adjusting for insulin‐stimulated glucose disposal rate (Galgani et al., 2008). These inconsistencies likely reflect, at least in part, heterogeneity in both the metabolic challenges applied and the metrics used to assess MetaFlex.

Classical assessments of MetaFlex rely on short‐duration indirect calorimetry (e.g., 15 to 60 min) performed under supraphysiological conditions, most commonly during a euglycemic‐hyperinsulinemic clamp (Kelley & Mandarino, 2000). While informative, these approaches capture only a narrow temporal window and may fail to reflect the dynamic regulation of substrate utilization under more physiological conditions (e.g., sleep RQ variability or sleep RQ nadir). To address these limitations, we previously proposed an integrative framework in which MetaFlex is conceptualized as the coordinated response of a metabolic *stressor* (e.g., dietary or exercise challenge), a *regulator* (insulin dynamics), and an *effector* (substrate oxidation), quantified using post‐challenge insulin variance and RQ variance (Rynders et al., 2018).

Measurement duration appears critical for capturing MetaFlex. For example, changes in substrate utilization may only emerge several hours after a metabolic challenge (Rudwill et al., 2018), suggesting that brief measurements may underestimate metabolic responsiveness. Whole‐room calorimetry offers a unique opportunity to capture continuous, minute‐by‐minute substrate oxidation over extended periods (12–48 h), such as 24‐h RQ dynamics and nocturnal RQ (RQ_sleep_). In particular, RQ_sleep_ and its nadir after a meal challenge may better reveal interindividual differences in substrate oxidation than clamp‐derived measures (McDougal et al., 2020, 2025; Zhang et al., 2021), as sleep represents a prolonged, behavior‐free postprandial state that amplifies differences, particularly in fat oxidation, after a high‐fat meal challenge (McDougal et al., 2025).

Beyond insulin‐clamp approaches, more physiological challenges, including dietary manipulations and acute exercise, have been proposed to assess MetaFlex (Galgani, Moro, & Ravussin, 2008). However, findings remain heterogeneous (Galgani & Fernández‐Verdejo, 2021). High‐carbohydrate meal challenges often fail to discriminate between metabolic phenotypes (Carnero et al., 2021). In contrast, acute high‐fat dietary challenges (single meal to 3 days, 50%–65% of energy from fat) have emerged as robust stressors for revealing impairments in MetaFlex (Begaye et al., 2020; de Souza Santos et al., 2016; Kardinaal et al., 2015; McDougal et al., 2020; Olenick et al., 2022), particularly when prolonged postprandial RQ measurements are considered (e.g., RQ_sleep_ or RQ_24h_), fat being a major contributor to day‐to‐day energy fluctuations (McDougal et al., 2020).

Acute exercise represents another potent metabolic challenge (Battista et al., 2021; Colpitts et al., 2021; Waldman et al., 2022). For example, sedentary individuals with obesity exhibit a greater relative increase in fat oxidation than in carbohydrate utilization during a graded submaximal exercise test, compared with lean or trained adults (Prior et al., 2014). Importantly, training status appears to modulate MetaFlex more strongly than body composition alone, as endurance‐trained individuals show greater MetaFlex than sedentary peers matched for BMI (Baugh et al., 2020; Carnero et al., 2021). These observations highlight the importance of accounting for both adiposity and training status when exercise is used as a metabolic stressor.

Given the diversity of metabolic challenges, populations, and outcome metrics across studies, direct comparisons remain difficult. In this ancillary analysis, we leveraged two completed whole‐room calorimetry studies to examine MetaFlex using the stressor‐regulator‐effector framework (Rynders et al., 2018) in response to two distinct physiologic stressors—short‐term dietary fat manipulation and acute exercise—using complementary indices (i.e., ΔRQ, 24‐h insulin and RQ variances, and RQ_sleep_) in adults with contrasting body weight and training statuses. We hypothesized that lean and trained individuals would display greater MetaFlex than untrained individuals with excess adiposity.

## 2 MATERIALS AND METHODS

Data from two previously published, independent human whole‐room calorimetry studies were used to examine the same overarching research question to compare 24‐h metabolic responses to metabolic challenges, that is, short‐term dietary fat manipulation (*Study 1*) and acute exercise (*Study 2*), in adults with contrasting weight status and/or training status. Participants were unique to each study, and data were analyzed separately within each study rather than pooled. Detailed protocols for *Study 1* (Bergouignan et al., 2012; Melanson et al., 2009) and *Study 2* (Bergouignan et al., 2014) have been published elsewhere. Only methods relevant to the present ancillary analyses are described below.

### 2.1 Institutional approval

*Study 1* was conducted between 2007 and 2009 (Bergouignan et al., 2012; Melanson et al., 2009), and S*tudy 2* between 2009 and 2011 (Bergouignan et al., 2014). Both studies were conducted in accordance with the Declaration of Helsinki and were approved by the Colorado Multiple Institutional Review Board (COMIRB) and the Scientific Advisory Board of the Clinical Translation Research Center (CTRC) at the University of Colorado‐Anschutz Medical Campus (CU‐AMC). Informed written consent was obtained from each study participant prior to participation.

### 2.2 Study participants

Participants in *Study 1* were male and female adults aged 20 and 45 years old and who were either healthy, lean and physically inactive (Ln‐Inactive, *n* = 6F/4M, body mass index—BMI, 19–25 kg/m^2^), healthy, lean and regular exercisers (Ln‐Trained, *n* = 6F/4M, BMI, 19–25 kg/m^2^), or with overweight or obesity and physically inactive (Ob‐Inactive, *n* = 4F/5M, BMI, 30–40 kg/m^2^; Table 1). Inclusion criteria were no history of prior obesity for the Ln‐Inactive and Ln‐Trained subjects and self‐reported inactive lifestyle (<1 exercise bout/week and sporting activities <1 h/week) for the Ln‐Inactive and Ob‐Inactive subjects. Ln‐Trained reported engaging in >5 h/week of endurance exercise at least over the 3 months prior to the study and actively participated in endurance athletic events within the year prior to the study.

**TABLE 1:** Study participant characteristics.

	*Study 1*			*Study 2*			
	Ln‐trained	Ln‐inactive	Ob‐inactive	Ln‐inactive	Ob‐inactive	RO‐inactive	RO‐trained
*N*	10 (6F/4M)	10 (6F/4M)	9 (4F/5M)	9 (4F/5M)	9 (5F/4M)	7 (4F/3M)	12 (6F/6M)
Age, years	30.8 ± 7.7	30 ± 8.0	37.7 ± 7.0	29.6 ± 8.0	34.8 ± 6.5	34.3 ± 8.8	33.3 ± 7.7
Weight, kg	59.9 ± 7.2	66.7 ± 7.6	111.0 ± 22.7	64.0 ± 10.0	98.3 ± 10.3	73.7 ± 10.0	81.9 ± 9.8
BMI, kg/m^2^	21.1 ± 1.3	22.5 ± 2.1	35.3 ± 4.3	21.5 ± 1.6	33.6 ± 2.5	26.9 ± 3.7	27.3 ± 2.8
FFM, kg	49.5 ± 7.8	48.9 ± 7.7	63.9 ± 11.4	48.9 ± 8.9	59.5 ± 10.6	50.8 ± 7.4	57.6 ± 10.7
BF, %	17.6 ± 5.0	26.6 ± 6.5	39.5 ± 3.3	22.5 ± 6.2	38.1 ± 7.3	30.0 ± 6.1	29.0 ± 9.1
VO_2peak_, L/min	2.9 ± 0.6	2.4 ± 0.6	2.6 ± 0.4	1.8 ± 0.4	1.6 ± 0.4	1.5 ± 0.5	1.2 ± 0.9
VO_2peak/FFM_, mL/min/kg	59.9 ± 3.8	47.5 ± 7.2	39.1 ± 4.0	37.4 ± 3.6	25.6 ± 4.6	27.7 ± 6.2	34.8 ± 10.0
HOMA‐IR	1.3 ± 1.9	1.89 ± 0.7	3.9 ± 1.9	2.4 ± 1.1	5.2 ± 4.0	3.7 ± 0.7	2.4 ± 1.1

*Note*: Values are mean ± standard deviation.

Abbreviations: BF, body fat; F, female; FFM, fat‐free mass; M, male; VO_2peak_, peak aerobic capacity; VO_2peak/FFM_, peak aerobic capacity normalized by FFM.

Participants in *Study 2* were male and female adults of 19–45 years old who were either healthy lean (Ln, *n* = 5F/4M, BMI, 19–25 kg/m^2^), with obesity (Ob, n = 5F/4M, BMI, 30–40 kg/m^2^), or who were weight‐reduced (*n* = 10F/9M, BMI, 25–40 kg/m^2^) (Table 1). Subjects with normal weight and obesity reported to be physically inactive (self‐reported <1 exercise bout/week or moderate‐to‐vigorous physical activity—MVPA <1 h/week). Weight‐reduced individuals were required to have lost at least 10% of their body weight and to maintain that weight loss for at least 6 months before the start of the study. Weight‐reduced individuals were further divided into two groups: those who reported having lost weight and maintained weight loss by dieting while being physically inactive (RO‐Inactive, *n* = 4F/3M; self‐reported <1 exercise bout/week or MVPA <1 h/week) and those who reported having lost weight and maintained weight loss by both dieting and exercising (RO‐Trained, *n* = 6F/6M; self‐reported moderate intensity exercise >30 min/session, >3 times/week).

Across both studies, exclusion criteria included cardiovascular or coronary artery disease, diabetes, thyroid or renal disease, smoking, medication use affecting weight or energy balance, or body weight change >5% within the preceding 6 months. Women were excluded if they planned to become pregnant, were lactating, were <6 months postpartum, or were postmenopausal.

### 2.3 Study design

*Study 1* was a randomized, three‐arm crossover trial examining the acute effects of a high‐fat diet and exercise on substrate oxidation. Each participant completed three 4‐day conditions separated by a 1–3 week washout period; in all conditions, total energy intake was estimated to meet energy needs to maintain energy balance (EB). The three conditions consisted of either a 4‐day low‐fat diet in resting conditions (LF‐EB, 65% carbohydrate—CHO, 20% fat, 15% protein of EI), a 2‐day LF‐EB diet followed by a 2‐day high‐fat diet in resting conditions (HF‐EB, 35% CHO, 50% fat, 15% protein of total EI), and a 4‐day LF‐EB diet coupled with an acute bout of moderate intensity exercise (55% of VO_2peak_) on day 4 (EX‐EB). Day 4 of each condition consisted of a 24‐h stay in a whole‐room calorimeter (WRC).

*Study 2* was a randomized, two‐arm crossover trial examining the effect of obesity and weight reduction on 24‐h substrate oxidation. Following a standardized 3‐day run‐in period (standard meal and no exercise), each participant spent 24 h in a WRC and was asked to either rest and receive energy intake to cover their energy needs (REST‐EB) or perform an acute bout of moderate‐intensity exercise on a cycle ergometer at 60% VO_2peak_ (EX‐ED) that was designed to induce an energy deficit equivalent to 15% of total energy expenditure. These calories were not replaced in the diet. Meals on both the 3‐day run‐in period and the 24 h study visit day were composed of 15% protein, 30% fat, and 55% CHO by energy intake. The two conditions were completed in random order and separated by a 1‐month washout.

In both studies, energy requirements were estimated as resting metabolic rate (RMR) × activity factor (1.4–1.9, depending on subjects' habitual physical activity, the condition, and the study day). RMR was calculated as the mean of measured RMR by indirect calorimetry and estimated RMR (23.9 × Fat‐Free Mass [FFM] + 372) based on dual‐energy x‐ray absorptiometry (DXA)‐derived FFM. Run‐in diet composition varied slightly (20% vs. 30% fat) across studies, reflecting design differences. All meals were prepared by the Clinical and Translational Research Center (CTRC) Metabolic Kitchen. Subjects were asked to eat all their food and bring any leftovers back for weighing.

### 2.4 Whole‐room calorimetry protocol

In both studies, participants reported to the CTRC on the morning of day 4 and entered the WRC at 0800 h for a 23‐h continuous measurement of energy expenditure and substrate oxidation. Urine was collected to estimate 24‐h protein utilization. Blood samples were collected fasting and 30–60 min after a meal through a leak‐free port in the calorimeter wall. Participants remained awake during daytime hours and performed standardized bench‐stepping bouts to mimic free‐living physical activity (three bouts in *Study 1*, two bouts in *Study 2*; 20 min at 72 steps/min). Lights were turned off at 2300 h in *Study 1* and at 2200 h in *Study 2*. Energy intake was provided as breakfast, lunch, dinner, and a light snack in *Study 1* at 0900 h, 1315 h, 1730 h, and 2000 h, and in *Study 2* at 0800 h, 1130 h, 1730 h, and 20 h00, respectively.

In *Study 1*, participants were asked to remain sedentary during the LF‐EB and HF‐EB conditions. During the EX‐EB condition, participants completed 1 h of cycling at 55% VO_2peak_ at 1000 h. In *Study 2*, participants remained sedentary during the REST‐EB condition and performed an exercise bout for 30 to 57 min at 60% of VO_2peak_ at 1600 h to induce an expenditure equivalent to 15% of their estimated total EE during the EX‐ED condition. In both studies, VO_2peak_ was assessed using a graded exercise test on a cycle ergometer or motorized treadmill and calculated as the average of the highest three 30‐s values, meeting at least two of the following criteria: (1) RQ >1.1, (2) heart rate within 10 beats/min of 85% of the age‐predicted maximum, and (3) an increase in oxygen absorption (VO_2_) in response to the final workload of 2.0 mL/kg/min.

### 2.5 24 h energy expenditure and substrate oxidation

Twenty‐three‐hour WRC data were extrapolated to 24‐h values using average‐minute data. Total EE and carbohydrate, fat, and protein oxidation were calculated from oxygen consumption (VO_2_), carbon dioxide production (VCO_2_), and urinary nitrogen excretion using standard equations (Jéquier et al., 1987). RQ was calculated as VCO_2_/VO_2_.

### 2.6 Blood analyses

Serum insulin concentrations were measured using a standard double‐antibody radioimmunoassay (Catalog number DSL‐1600, Diagnostic Systems Laboratory, Webster, CT).

### 2.7 Metabolic flexibility

MetaFlex was assessed using four complementary metrics:

1. ΔRQ_day‐night_, calculated as the difference between mean daytime RQ and mean RQ _sleep_.
2. RQ_sleep_, calculated as the mean nocturnal RQ (2300–0700 in *Study 1*; 2200–0700 in *Study 2*).
3. 24‐h RQ variance, calculated as the average squared deviation of each RQ value from the 24‐h mean RQ and
4. 24‐h insulin variance, calculated as the average squared deviation of each insulin value from the 24‐h mean insulin.

Daytime and nighttime periods were defined by clock time. Mathematical variance‐based indices were interpreted within the stressor‐regulator‐effector framework (Bergouignan et al., 2011; Rynders et al., 2018), whereby greater MetaFlex is characterized by high RQ variance, indicating a shift in substrate use despite low insulin variation, and metabolically inflexibility by low RQ variance coupled with high insulin variance, indicating a small shift in carbohydrate versus fat use despite large insulin fluctuations.

### 2.8 Statistical analysis

Data are presented as mean and standard error, unless otherwise stated. Twenty‐four‐hour RQ and insulin variances were not normally distributed and were therefore log‐transformed prior to analysis. Statistical inference was based on transformed data, while descriptive statistics, least square means (LSMeans), and figures are reported on the original units for physiological interpretability. Linear mixed models were used with group (*Study 1*: Ln‐Inactive, Ln‐Trained, Ob‐Inactive; *Study 2*: Ln‐Inactive, Ob‐Inactive, RO‐Inactive, RO‐Trained), condition (*Study 1*: LF‐EB, HF‐EB, EX‐EB; *Study 2*: REST‐EB, EX‐ED), and group‐by‐condition interaction as fixed effects and subjects as random effects. A compound symmetry covariance structure was applied with degrees of freedom estimated using the Kenward‐Roger method. For *Study 1*, the calorimeter version (old vs. new) was included as a covariate to account for the relocation of the research facilities. Sensitivity analyses evaluated fat mass, insulin, and/or cardiorespiratory fitness (VO_2peak_) as potential confounders. Statistical significance was set at *p* < 0.05 for main effects and *p* < 0.1 for interactions. All the analyses were run on SAS version 9.4 (Cary, NC: SAS Institute Inc.).

## 3 RESULTS

### 3.1 Participant characteristics

Baseline demographic, anthropometric, and metabolic characteristics of *Study 1* and *Study 2* participants are presented in Table 1.

### 3.2 Short‐term high‐fat feeding alters variance‐based MetaFlex indices independent of group (study 1)

**FIGURE 1:**
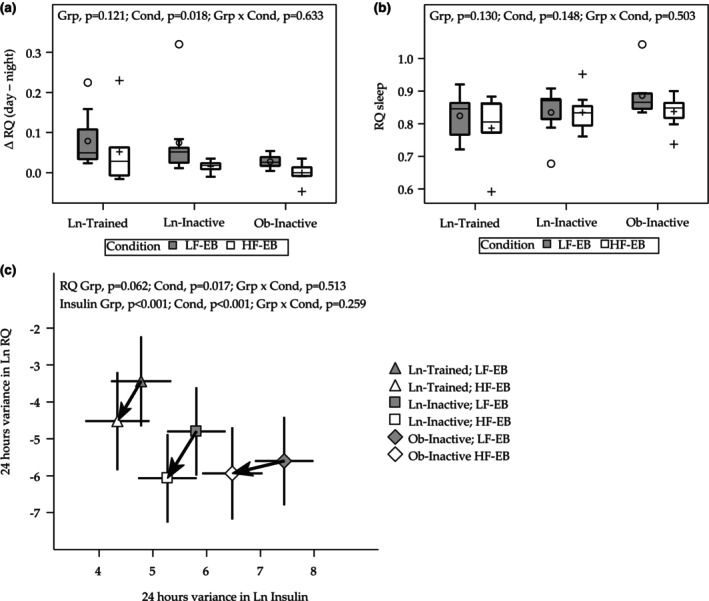
Metabolic Flexibility metrics under low‐fat and high‐fat dietary conditions in adults with contrasting training and adiposity statuses. (a) Boxplots showing the difference in respiratory quotient (RQ) between day and night in physically inactive (Ln‐Inactive; *n* = 6F/4M, BMI 19–25 kg/m^2^), lean, regularly exercising (Ln‐Trained; *n* = 6F/4M, BMI 19–25 kg/m^2^), and overweight/obese, physically inactive (Ob‐Inactive; *n* = 4F/5M, BMI 30–40 kg/m^2^) groups following two dietary conditions: 4 days of low‐fat diet (LF‐EB) and 2 days of LF‐EB followed by 2 days of high‐fat diet (HF‐EB); (b) Boxplots of nighttime RQ (RQ_sleep_) between 23:00 and 07:00 under LF‐EB and HF‐EB; and (c) Scatterplot illustrating the relationship between 24‐h RQ variance and 24‐h insulin variance for the same groups under LF‐EB and HF‐EB. In figure c, individuals positioned in the upper‐left quadrant (high RQ variance, low insulin variance) exhibit greater metabolic flexibility compared to those in the lower‐right quadrant (low RQ variance, high insulin variance). Black arrows indicate the integrated change in both 24‐h RQ and insulin variance in response to HF‐EB versus LF‐EB; arrows pointing in the same direction reflect similar MetaFlex responses.

In *Study 1*, we found that ΔRQ_day‐night_ significantly increased from the LF‐EB to the HF‐EB condition (Difference LSMeans ± SE, 0.039 ± 0.016, *p* = 0.018; Figure 1a), showing a modest but significant effect of dietary fat on MetaFlex. No group effect was detected, suggesting that variations in dietary fat could serve as a stressor for evaluating MetaFlex across different fitness levels and body compositions. RQ_sleep_ did not differ between dietary conditions (0.027 ± 0.018, *p* = 0.1475; Figure 1b) and showed no group effect.

In contrast, variance‐based indices were strongly affected by the dietary challenge. Compared with LF‐EB, HF‐EB was associated with greater 24‐h RQ variance (0.895 ± 0.345, *p* = 0.02; Figure 1c) and lower 24‐h insulin variance (0.650 ± 0.132, *p* < 0.001; Figure 1c) across groups. These effects remained significant after adjustment for fat mass, fasting insulin, and/or VO_2peak_, indicating that the dietary manipulation was the primary driver of the variance response.

Training and adiposity status mainly differentiated 24‐h insulin variance, with consistent between‐group differences under both LF‐EB and HF‐EB. Under the LF‐EB condition, insulin variance was lower in Ln‐Trained compared to Ln‐Inactive (−1.029 ± 0.382, *p* = 0.011) and Ob‐Inactive (−2.664 ± 0.383, *p* < 0.001). In addition, Ob‐Inactive showed higher variance than Ln‐Inactive (1.635 ± 0.378, *p* = 0.001).

A similar pattern was observed under HF‐EB. Ln‐Trained showed lower insulin variance than Ln‐Inactive (−0.931 ± 0.397, *p* = 0.025) and Ob‐Inactive (−2.138 ± 0.403, *p* < 0.0001). While Ln‐Inactive also showed lower variance than Ob‐Inactive (−1.207 ± 0.385, *p* = 0.004). Adjusting for baseline insulin values did not modify the between‐group comparisons. No group‐by‐condition interactions were detected for any outcome, indicating that group and dietary condition contributed independently.

### 3.3 Acute exercise performed in energy balance fails to reveal a differential metabolic flexibility response (study 1)

**FIGURE 2:**
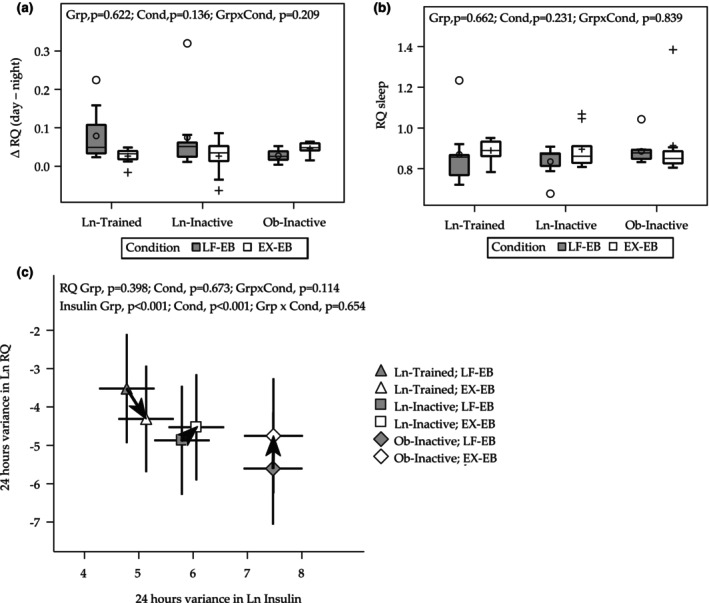
Metabolic Flexibility Metrics in response to an acute exercise versus resting conditions in individuals with contrasting adiposity and training statuses. (a) Boxplots showing the difference in respiratory quotient (RQ) between day and night under two conditions: Low‐fat diet (LF‐EB) and an exercise bout (EX‐EB) in lean, physically inactive (Ln‐Inactive; *n* = 6F/4M, BMI 19–25 kg/m^2^), lean, regularly exercising (Ln‐Trained; *n* = 6F/4M, BMI 19–25 kg/m^2^), and overweight/obese, physically inactive (Ob‐Inactive; *n* = 4F/5M, BMI 30–40 kg/m^2^)groups. (b) Boxplots of average nighttime RQ (RQ_sleep_) measured between 23:00 and 07:00 under LF‐EB and EX‐EB; and (c) Scatterplots depicting the relationship between 24‐h RQ variance and 24‐h insulin variance for the same groups under LF‐EB and EX‐EB. In figure c, black arrows indicate the directional change in the MetaFlex index between LF‐EB and EX‐EB, defined by the relationship between RQ and insulin variances. A higher 24‐h RQ variance combined with a lower 24‐h insulin variance reflects greater MetaFlex.

When acute exercise was performed in energy balance (LF‐EB vs. EX‐EB), no condition effects were observed for ΔRQ_day‐night_, RQ_sleep_, 24‐h RQ variance, and 24‐h insulin variance (Figure 2a–c). Similarly, ΔRQ_day‐night_, RQsleep, and 24‐h RQ variance did not differ between groups.

A significant group effect was observed for 24‐h insulin variance (*p* < 0.001). Ln‐Trained had higher 24‐h insulin variance than Ln‐Inactive (Difference LSMeans ± SE, −0.969 ± 0.030, *p* = 0.004) and Ob‐Inactive (−2.517 ± 0.312, *p* < 0.001). In addition, Ln‐Inactive had lower insulin variance than Ob‐Inactive (−1.548 ± 0.312, *p* < 0.001). However, these between‐group differences were no longer significant after adjustment for fasting insulin levels, suggesting that baseline insulin largely accounted for the observed group separation in response to EX‐EB. No significant group‐by‐condition interactions were noted.

### 3.4 Exercise‐induced energy deficit unmasks group differences in insulin dynamics (study 2)

**FIGURE 3:**
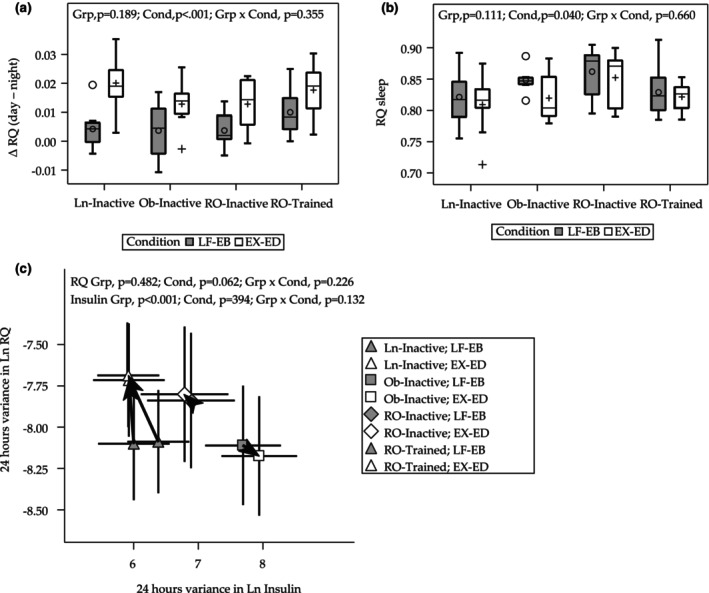
Metabolic Flexibility Metrics in response to an Acute Exercise Bout coupled with Energy Deficiency in individuals with contrasting weight and training statuses. (a) Boxplots showing the difference in respiratory quotient (RQ) between day and night under two conditions: Resting in energy balance (REST‐EB), and an exercise bout (EX‐EB) in healthy lean (Ln‐Inactive; *n* = 5F/4M, BMI 19–25 kg/m^2^), individuals with obesity (Ob‐Inactive; *n* = 5F/4M, BMI 30–40 kg/m^2^), those self‐reporting physical inactivity (RO‐Inactive; *n* = 4F/3M; <1 exercise bout/week or <1 h/week of moderate‐to‐vigorous activity), and individuals who reported weight loss maintenance through diet and regular exercise (RO‐Trained; *n* = 6F/6M; moderate‐intensity exercise >30 min/session, >3 times/week). (b) Boxplots of average nighttime RQ (RQ_sleep_) measured between 23:00 and 07:00 under REST‐EB and EX‐EB; and (c) Scatterplots illustrating the relationship between 24‐h RQ variance and 24‐h insulin variance for the same groups under REST‐EB and EX‐EB. In Figure c, black arrows indicate the directional change in the MetaFlex index between REST‐EB and EX‐EB, defined by the relationship between RQ and insulin variances. A higher 24‐h RQ variance combined with a lower 24‐h insulin variance reflects greater MetaFlex.

In *Study 2* (Ln‐Inactive, Ob‐Inactive, RO‐trained, and RO‐Inactive), acute exercise designed to induce an approximate 15% energy deficit (EX‐ED) significantly reduced ΔRQ_day‐night_ compared with the REST‐EB (Difference LSMeans ± SE: −0.011 ± 0.002, *p* < 0.001; Figure 3a). However, after adjustment for individual VO_2peak_ and fat mass, between‐group differences were not significant (*p* > 0.05), indicating that variations in ΔRQ were primarily influenced by fitness and adiposity. RQ_sleep_ was also modestly affected by acute exercise challenge with energy deficit compared to the LF‐EB condition (0.014 ± 0.007, *p* = 0.039, Figure 3b), with no group effect.

No significant group effect (*p* = 0.482) or condition effect (−0.189 ± 0.098, *p* = 0.06) was observed for 24‐h RQ variance (Figure 3c). In contrast, 24‐h insulin variance differed by group but not by condition (Figure 3c). Ob‐Inactive exhibited higher insulin variance than Ln‐Inactive (−1.852 ± 0.366, *p* < 0.0001), RO‐Trained (1.667 ± 0.344, *p* < 0.001), and RO‐Inactive (0.979 ± 0.0.407, *p* = 0.022). In addition, RO‐Inactive had a higher 24‐h insulin variance than Ln‐Inactive (−0.872 ± 0.340, *p* = 0.036), whereas RO‐Trained and RO‐Inactive did not differ. No group‐by‐condition interactions were detected, indicating that group and condition independently affected the different MetaFlex metrics.

## 4 DISCUSSION

We examined the changes in ΔRQ_day‐night_, RQ_sleep_, and mathematically calculated variances of 24‐h RQ and insulin in response to three acute metabolic challenges (high‐fat diet in energy balance and acute exercise performed in energy balance or under energy deficit) to assess the ability of the body to adjust substrate oxidation to fuel availability and energy demand, that is, MetaFlex, in adults with contrasting body weight and training status. Overall, our findings indicate that MetaFlex is both challenge‐ and metric‐dependent, and that variance‐based indices provide complementary information beyond traditional ΔRQ‐derived metrics under physiologically relevant dietary and energetic perturbations.

When exposed to a short‐term, high‐fat diet under energy balance, participants exhibited clear shifts in variance‐based MetaFlex indices across groups, despite no group differences in ΔRQ_day‐night_ or RQ_sleep_. Specifically, high‐fat feeding increased 24‐h RQ variance while reducing 24‐h insulin variance in all groups, indicating enhanced substrate switching with lower insulin fluctuation. Group differences emerged primarily in 24‐h insulin variance, with individuals with obesity exhibiting greater fluctuations in insulin concentration than lean participants, regardless of training, suggesting reduced efficiency in insulin‐mediated metabolic adaptation. These observations support the notion that dietary fat manipulation represents a physiologically relevant stressor for probing MetaFlex, even in the absence of an energy imbalance.

ΔRQ has long been considered a reference metric for measuring MetaFlex, reflecting the ability to switch from lipid oxidation in the fasted state to carbohydrate oxidation under insulin‐stimulated conditions. In the present study, however, ΔRQ_day‐night_ did not discriminate between groups in response to any of the tested challenges, including high‐fat feeding in energy balance, exercise in energy balance, or exercise‐induced energy deficit, despite marked differences in body weight and training status.

Previous studies have reported impaired ΔRQ responses following graded exercise in individuals with severe obesity (BMI, 39.0–54.0 kg/m2) and altered glycemic control, including prediabetes and type 2 diabetes, compared with individuals with lower body weight and normal glycemic control (Battista et al., 2021). The discrepancy with our findings may reflect differences in metabolic health status, as our participants with obesity were free of overt dysglycemia. Previous studies primarily assessed responses during acute exercise bouts, whereas the current analysis is based on 24‐h calorimeter measurements, which may provide a more comprehensive and physiologically representative assessment and thereby help explain the apparent contradictory findings. Collectively, these data suggest that ΔRQ may primarily detect more advanced metabolic impairments and may lack sensitivity to detect more subtle or early alterations in MetaFlex across heterogeneous but metabolically healthy populations.

Contrary to expectations, RQ_sleep_ did not differ between conditions or groups. This contrasts with prior work reporting lower nighttime RQ or RQ nadir values in metabolically flexible individuals (Carnero et al., 2021; Zhang et al., 2021). For instance, Zhang et al. observed lower sleep nadir RQ in metabolically flexible individuals than in metabolically inflexible individuals, reflecting greater reliance on lipid oxidation during sleep (Zhang et al., 2021). Carnero et al. reported lower RQ_sleep_ in lean‐trained individuals compared with sedentary adults with obesity despite similar ΔRQ responses (Carnero et al., 2021). Of note, these studies did not incorporate tightly controlled regulation of energy balance, which could explain the lack of significant results, as we show that energy deficit might be advantageous when supporting metabolic flexibility assessment to exercise as a stressor.

Several factors may explain the absence of group differences in our study, including limited statistical power, heterogeneity in metabolic phenotypes, and methodological choices. We defined RQ_sleep_ using a broad nocturnal window (2300–0700) rather than a narrower window or the nadir of RQ_sleep_, prioritizing signal robustness over sensitivity. While this approach reduces noise, it may attenuate the detection of subtle differences. Taken together, these observations suggest that RQ_sleep_ remains a promising complementary MetaFlex assessment, but standardized definitions and analytical approaches are needed to optimize its interpretability and reproducibility.

Because MetaFlex reflects dynamic regulation rather than static substrate use, we further examined variance‐based indices 24‐h RQ and insulin to capture the interaction between substrate oxidation and hormonal regulation (Bergouignan et al., 2012; Rynders et al., 2018). Unlike ΔRQ and RQ_sleep_, these indices revealed more consistent patterns across metabolic challenges. Following high‐fat feeding, in terms of energy balance, lean individuals—both trained and inactive—exhibited higher 24‐h RQ variance and lower 24‐h insulin variance than individuals with obesity, consistent with more efficient metabolic regulation. Importantly, variance‐based indices should not be interpreted as markers of inflexibility in isolation. Within the stressor‐regulator‐effector framework, higher RQ variance in the context of lower insulin variance reflects greater metabolic responsiveness achieved with reduced regulatory demand. The consistent separation observed across dietary conditions suggests that these dynamic metrics capture a key dimension of MetaFlex that is not accessible through RQ metrics alone.

When exercise was performed under energy balance, none of the MetaFlex indices – including variance‐based measures‐ were altered, indicating that acute moderate‐intensity exercise without accompanying energy deficit is insufficient to reveal phenotypic differences in MetaFlex. In contrast, when exercise induced a 15% energy deficit, group differences emerged in 24‐h insulin variance, with lean inactive and reduced‐obese trained individuals displaying lower insulin variability than reduced‐obese inactive individuals. These findings indicate that MetaFlex is more readily unmasked when exercise is combined with an energetic challenge, rather than exercise per se. This interpretation is consistent with previous research showing reduced RQ variance in adults with higher BMI during daily activities (e.g., sitting, standing, and transitions) (Júdice et al., 2021) and greater exercise‐induced substrate flexibility in lean‐trained versus sedentary women with obesity (Waldman et al., 2022). However, further work is needed to establish a standard exercise metabolic challenge that optimally probes MetaFlex under physiological conditions.

Our findings also inform the longstanding debate regarding fitness versus fatness in metabolic health (Barry et al., 2014). In line with previous intervention studies (Fechner et al., 2020; Galgani & Fernández‐Verdejo, 2021; Goldenshluger et al., 2021; Goodpaster et al., 2003; Martins et al., 2022; Ooi et al., 2021), individuals who had lost weight through a combination of dietary restriction and regular exercise displayed MetaFlex profiles comparable to those of lean individuals, whereas reduced‐obese inactive individuals remained less flexible. These results suggest that training is needed in addition to weight loss to offset the adverse effects of higher body weight on MetaFlex.

Limitations should be acknowledged. Sample sizes were modest, limiting statistical power and precluding evaluation of sex‐specific effects, which are likely relevant for MetaFlex (Zhang et al., 2021). In addition, the absence of an obese‐trained group limited in *study 1* and lean‐trained in *study 2* our ability to fully disentangle the independent effects of adiposity and training status.

## 5 CONCLUSIONS

MetaFlex is not a unitary construct and cannot be fully captured by a single metric or metabolic challenge. Our findings suggest that MetaFlex detection critically depends on both the nature of the applied metabolic stressor and the outcome metric used. Under energy‐balance conditions, short‐term high‐fat feeding is more effective than acute exercise in revealing MetaFlex differences, whereas coupling acute exercise with an energy deficit enables the detection of between‐group differences. Individuals with obesity exhibit reduced MetaFlex compared with lean individuals, while weight loss combined with training is associated with partial restoration of MetaFlex. Further studies integrating 24‐h insulin dynamics, RQ variances, and sleep‐specific substrate oxidation are warranted to refine physiological assessments of MetaFlex and its implication for metabolic health.

## AUTHOR CONTRIBUTIONS

**Carmen P. Ortega‐Santos:** Formal analysis. **Edward L. Melanson:** Conceptualization; data curation; funding acquisition; investigation; methodology. **Daniel H. Bessesen:** Conceptualization; data curation; funding acquisition; investigation; methodology; project administration; supervision. **Pan Zhaoxing:** Formal analysis. **Corey A. Rynders:** Formal analysis. **Audrey Bergouignan:** Conceptualization; data curation; investigation; methodology; supervision.

## FUNDING INFORMATION

Study 1 was supported by a Mentored Scientist Award (National Institutes of Health K01‐DK‐061348, DK123334) to E. L. Melanson, the University of Colorado Denver Clinical and Translation Science Award (1UL1‐RR‐025780). Study 2 was supported by the NIH/NCRR Colorado CTSI Grant: UL1 RR025780 and the Colorado Nutrition Obesity Research Center (P30DK048520).

## CONFLICT OF INTEREST STATEMENT

The authors declare no conflicts of interest.

## ETHICS STATEMENT

The study was conducted in accordance with the Declaration of Helsinki and approved by the Institutional Review Board of the University of Colorado‐Anschutz Medical Campus (*Study 1* was conducted between 2007 and 2009, and *Study 2* was conducted from 2009 to 2011).

## INFORMED CONSENT STATEMENT

Informed consent was obtained from all subjects involved in the study.

## Data Availability

The datasets generated during and/or analyzed during the current study are available from the corresponding author on reasonable request.
